# Exposure to Bisphenol-A during Pregnancy Partially Mimics the Effects of a High-Fat Diet Altering Glucose Homeostasis and Gene Expression in Adult Male Mice

**DOI:** 10.1371/journal.pone.0100214

**Published:** 2014-06-24

**Authors:** Marta García-Arevalo, Paloma Alonso-Magdalena, Junia Rebelo Dos Santos, Ivan Quesada, Everardo M. Carneiro, Angel Nadal

**Affiliations:** 1 Instituto de Bioingeniería, Universidad Miguel Hernández de Elche, Elche, Spain; 2 Departamento de Biología Aplicada, Universidad Miguel Hernández de Elche, Elche, Spain; 3 Centro de Investigación Biomédica En Red de Diabetes y Enfermedades Metabólicas Asociadas, CIBERDEM, Universidad Miguel Hernández de Elche, Elche, Spain; 4 Departamento de Biologia Estructural e Funcional, Instituto de Biologia, Universidade Estadual de Campinas, UNICAMP, Campinas, Brazil; University of Cordoba, Spain

## Abstract

Bisphenol-A (BPA) is one of the most widespread EDCs used as a base compound in the manufacture of polycarbonate plastics. The aim of our research has been to study how the exposure to BPA during pregnancy affects weight, glucose homeostasis, pancreatic β-cell function and gene expression in the major peripheral organs that control energy flux: white adipose tissue (WAT), the liver and skeletal muscle, in male offspring 17 and 28 weeks old. Pregnant mice were treated with a subcutaneous injection of 10 µg/kg/day of BPA or a vehicle from day 9 to 16 of pregnancy. One month old offspring were divided into four different groups: vehicle treated mice that ate a normal chow diet (Control group); BPA treated mice that also ate a normal chow diet (BPA); vehicle treated animals that had a high fat diet (HFD) and BPA treated animals that were fed HFD (HFD-BPA). The BPA group started to gain weight at 18 weeks old and caught up to the HFD group before week 28. The BPA group as well as the HFD and HFD-BPA ones presented fasting hyperglycemia, glucose intolerance and high levels of non-esterified fatty acids (NEFA) in plasma compared with the Control one. Glucose stimulated insulin release was disrupted, particularly in the HFD-BPA group. In WAT, the mRNA expression of the genes involved in fatty acid metabolism, *Srebpc1*, *Pparα* and *Cpt1β* was decreased by BPA to the same extent as with the HFD treatment. BPA treatment upregulated *Pparγ* and *Prkaa1* genes in the liver; yet it diminished the expression of *Cd36*. Hepatic triglyceride levels were increased in all groups compared to control. In conclusion, male offspring from BPA-treated mothers presented symptoms of diabesity. This term refers to a form of diabetes which typically develops in later life and is associated with obesity.

## Introduction

Bisphenol-A BPA is a high production volume chemical, which is widely used in manufacturing polycarbonate plastic and epoxy resins. Releases of BPA to the environment are higher than 1 million pounds per year (Environmental Protection Agency; http://www.epa.gov/oppt/existingchemicals/pubs/actionplans/bpa.html). BPA is found in many dairy products, including food packaging, i.e: inner lining of cans, baby bottles, thermal paper, etc. [1]. When polycarbonate plastic is heated or treated with acid or basic media the ester bonds formed between each BPA monomer are hydrolyzed and BPA then migrates [2]. Because of its widespread use, BPA is found in the urine of 93% of US citizens [3] and its concentrations in urine and blood are as high as 1–18 nM [4].

Bisphenol-A exerts its actions by interfering with the endocrine system at different levels, although its role as an estrogen mimic is the best known one [5]. Using genetically modified mice, it has been demonstrated that it acts as a potent estrogenic compound after binding to extranuclear estrogen receptors, ERα and ERβ [6], [7], [8].

The etiology of type-2 diabetes mellitus (T2DM) is based on the decreased insulin sensitivity in peripheral tissue together with the disruption of pancreatic β-cell mass and function. Both genetic and environmental factors play key roles. Epidemiology has established robust associations of T2DM with obesity, age, sedentary life, economic status, ethnicity, smoking and exposure to endocrine disruptors [9], [10]. Exposure to bisphenol-A has been epidemiologically associated to T2DM, cardiovascular diseases, insulin resistance and obesity [11], [12], [13].

Animal studies indicate that exposure to BPA is involved in developmental alterations of the reproductive tract and nervous system, the incidence of cancer and also in metabolic disorders including type-2 diabetes and obesity [10], [14], [15], [16]. The timing of exposure is a key factor to obtain different metabolic phenotypes. In adult animals, the exposure to BPA provokes decreased food intake, lower body temperature and locomotive activity, glucose intolerance, insulin resistance and alterations of pancreatic beta cell function [17], [18]. Perinatal exposure elicits increased body weight, adiposity and alterations in glucose tolerance and insulin sensitivity [19], [20], [21], [22]. A recent study indicates that the perinatal exposure of rats to low doses of BPA increases weight gain and exacerbates the effect of a high fat diet on glucose homeostasis and β-cell function [20]. In mice, exposure to BPA during pregnancy induced hyperinsulinaemia, glucose intolerance and insulin resistance in 6 month old male offspring [23], [24].

In our research, pregnant mice were exposed to a vehicle or BPA 10 µg/kg/day and one month old male offspring were fed either a normal diet (ND) or a high fat diet (HFD) for 13 or 24 weeks. We then studied body weight change, glucose homeostasis, glucose stimulated insulin secretion (GSIS) and insulin content as well as gene expression in active metabolic tissue: white adipose tissue, liver and skeletal muscle.

## Materials and Methods

### Animals and treatment

Pregnant OF-1 mice were purchased from Charles River (Barcelona, Spain) and individually housed under standard conditions. Mice were maintained on 2014 Teklad Global 14% Protein Rodent Maintenance Diet (Harlan Laboratories, Barcelona, Spain), which does not contain alfalfa or soybean meal (chow diet). The composition of the diet is as follows: calories from protein, 18%; calories from fat, 11%; and calories from carbohydrate, 71%, with energy of 2.9 kcal/g.

The ethical committee of Miguel Hernandez University “Comisión de Ética en la Investigación Experimental” specifically reviewed and approved this study (approval ID: IB-AN-001-11). Animals were treated humanely and with regard to alleviate suffering.

BPA was dissolved in tocopherol-stripped corn oil and administered subcutaneously on days 9–16 of gestation. The daily dose used was 10 µg/kg. Pups of the same treatment group were pooled together and then placed in equal numbers with foster mothers of the same treatment group. Animals were weaned on postnatal day 21 and housed (8 mice/group) from weaning through adulthood in type III cage of polycarbonate plastic (Tecniplast), 530 cm^2^. We used new cages to avoid BPA release as much as possible. Pups housed together were of the same sex. After the age of one month, they were maintained, ad libitum, during 13 or 24 weeks on a chow diet or High Fat Diet (HFD), D12451, (Research Diets Inc., New Brunswick, NJ, USA.). Experiments were performed when mice were 17 or 28 weeks of age. The composition of the diet is as follows: calories from protein, 20%; calories from fat, 45%, and calories from carbohydrate, 35%, with energy of 4.7 kcal/g. We should consider that, minor differences in the composition exist between a chow diet and the corresponding control low-fat diet for the HFD. Composition of diets may determine changes in gene expression, however, differences between chow diet and HFD or control low-fat diet and HFD are low.

Only male offspring was used in this study since previous work from our group demonstrated that under the same BPA treatment no differences on glucose homeostasis were found in the female offspring, at least during the analyzed period of life.

### Islet cell isolation

Pancreatic islets of Langerhans were isolated by collagenase (Sigma, Madrid, Spain) digestion (modified from [25]). The solution used for the isolation of the islets of Langerhans contained (in mmol/l): 115 NaCl, 10 NaHCO_3_, 5 KCl, 1.1 MgCl_2_, 1.2 NaH_2_PO_4_, 2.5 CaCl_2_, 25 HEPES, and 5 D-glucose, pH 7.4, as well as 0.25% BSA. Freshly isolated islets were used for calcium and insulin secretion measurements after 2 hours of recovery.

### Glucose and insulin tolerance tests

For intraperitoneal glucose tolerance tests (ipGTT), animals were fasted for 6 h [26], and blood samples were obtained from the tail vein. Animals were then injected intraperitoneally with 1.5 g/kg body weight of glucose, and blood samples were taken at the indicated intervals.

For intraperitoneal insulin tolerance tests (ipITT), fed animals were used. Animals were injected intraperitoneally with 0.75 IU/kg body weight of soluble insulin, and blood samples were obtained from the tail vein. Blood glucose was measured in each sample using an Accu-check compact glucometer (Roche, Madrid, Spain). Levels of glycemia after insulin injection are expressed as % of glycemia compared to basal glycemia levels in fed state.

ipGTT and ipITT were made at 17 and 28 weeks of age.

### Serum analysis

Blood samples were collected for biochemical analysis at decapitation in fed state animals. Serum samples were obtained by centrifugation for 15 minutes at 1200 rpm at 4°C. Samples were stored at −80°C. The serum insulin level was analyzed by Ultra Sensitive Mouse Insulin ELISA Kit (Crystal Chem, Downers Grove, IL). Serum glycerol and triglyceride levels were measured by Serum Triglyceride Determination kit (TR0100, Sigma). Non-esterified fatty acids (NEFAs) were measured using a NEFA-HR(2) kit for serum determination (Wako).

### Insulin secretion and content

Freshly isolated islets were left to recover in the isolation medium for 2 h in the incubator at 37°C. After recovery, groups of 5 were transferred to 400 µl of a buffer solution containing 140 mM NaCl, 4.5 mM KCl, 2.5 mM CaCl_2_, 1 mM MgCl_2_, 20 mM HEPES and the corresponding glucose concentration (3, 8 or 16 mM) with final pH at 7.4. Afterwards, 100 µl of the buffer solution with the corresponding glucose concentration with 5% BSA was added, incubated at room temperature for 3 min and left to cool down for 15 min on ice. Then, the medium was collected and insulin was measured in duplicate samples by radioimmunoassay using a Coat-a-Count kit (Siemens, Los Angeles, CA, USA). Protein concentration was measured by the Bradford dye method [27].

To obtain the insulin content, the islets grouped in batches of 5 were handpicked with a micropipette and incubated overnight in an ethanol/HCl buffer (75% Ethanol (^v^/_v_); 0.4% HCl (stock 37%) (^v^/_v_) and 24.6% distilled water (^v^/_v_)) at 4°C. At the end of the incubation period, the buffer was removed and studied for insulin content using radioimmunoassay with a Coat-a-Count kit. Protein determination was performed using the Bradford dye method. Insulin and protein content was determined in each islet sample and the ratio of both parameters was calculated for each sample.

### Real-time PCR

Quantitative PCR assays were performed using CFX96 Real Time System (Bio-Rad, Hercules, CA) and 7500 Real Time PCR System (Applied Biosystems, Foster City, CA). RNA extraction was made with TriPure Isolation Reagent (Roche), and 1 µg of RNA was used for retrotranscription reaction (HighCapacity cDNA Reverse transcription, Applied Biosystems). Reactions were carried out in a final volume of 10 µl, containing 200 nM of each primer, 1 µl of cDNA, and 1×IQ SYBR Green Supermix (Bio-Rad). Samples were subjected to the following conditions: 10 min at 95°C, 40 cycles (10 s at 95°C, 7 s at 60°C, and 12 s at 72°C), and a melting curve of 63–95°C with a slope of 0.1 C/s. The housekeeping gene was ribosomal protein large P0, also known as 36B4, and it was used as the endogenous control for quantification [28]. The resulting values were analyzed with CFX Manager Version 1.6 (Bio-Rad), and values were expressed as the relative expression respect to control levels (2^−ΔΔCT^) [29]. Primers are described in Table S1.

### Triglycerides extraction and quantification in liver

Liver tissue (100 mg) was homogenized and then added 500 µL of SDS 0.1% in an eppendorf tube. The mixture was homogenized again and was left shaking overnight at room temperature. Then 400 µL of the mix were transferred to a new tube, a volume of methanol was added and the sample was mixed. After that, 800 µL of chloroform were added and the mixture was incubated during 30 min on ice. Then 48 µL of KCl 0.5 M were added and the mixture was incubated during 30 min on ice. The sample was then centrifuged for 10 min at 2000 rpm at 4°C. The organic phase was transferred to an eppendorf tube and the chloroform was let to evaporate overnight. Finally the extract was resuspended with 100% Ethanol and triglycerides were measured by using the Triglyceride Determination kit (TR0100, Sigma).

### Recording intracellular calcium concentrations

Freshly isolated islets of Langerhans were loaded with 5 µM Fura-2 AM for at least 1 h at room temperature. Calcium recordings in islets were obtained by imaging intracellular calcium under an inverted epifluorescence microscope (Zeiss, Axiovert 200). Images were acquired every 2 s with an extended Hamamatsu Digital Camera C4742-95 (Hamamatsu Photonics, Barcelona, Spain) using a dual filter wheel (Sutter Instrument CO, CA, USA) equipped with 340 nm and 380 nm, 10 nm bandpass filters (Omega optics, Madrid, Spain). Data was acquired using Aquacosmos software from Hamamatsu (Hamamatsu Photonics, Barcelona, Spain). Fluorescence changes are expressed as the ratio of fluorescence at 340 nm and 380 nm (F340/F380). Results were plotted and analyzed using commercially available software (Sigmaplot, Jandel Scientific).

### Statistical analysis

SigmaStat 3.1 software (Systat Software, Inc., Chicago, IL, USA) was used for all statistical analyses. To assess differences between treatment groups for each exposure paradigm, we used the one-way analysis of variance (ANOVA) followed by the Holm-Sidak or Tukey method. When data did not pass the parametric test, we used ANOVA on ranks followed by Dunn's test. Results were considered significant at p<0.05. Data are shown as mean ± SEM.

## Results

### The effects of BPA on weight, food intake, retroperitoneal and gonadal fat pad weight and plasma NEFA

To examine the effects of BPA on the glucose metabolism of offspring, we treated pregnant mice with either a vehicle or BPA at a dose of 10 µg/kg/day on GD9–GD16. Throughout the groups, animals were matched for gestation day to minimize potential differences in the background levels of maternal hormones during the last phase of pregnancy. One month old mice were exposed to a normal chow diet (ND) or a high fat diet (HFD) for 13 weeks or 24 weeks. In total, we had 4 different groups for each age: vehicle treated animals fed ND (Control), animals exposed to 10 µg/kg/day of BPA fed ND (BPA), vehicle treated animals fed a HFD (HFD) and animals exposed to 10 µg/kg/day of BPA fed a HFD (HFD-BPA).

**Figure 1:**
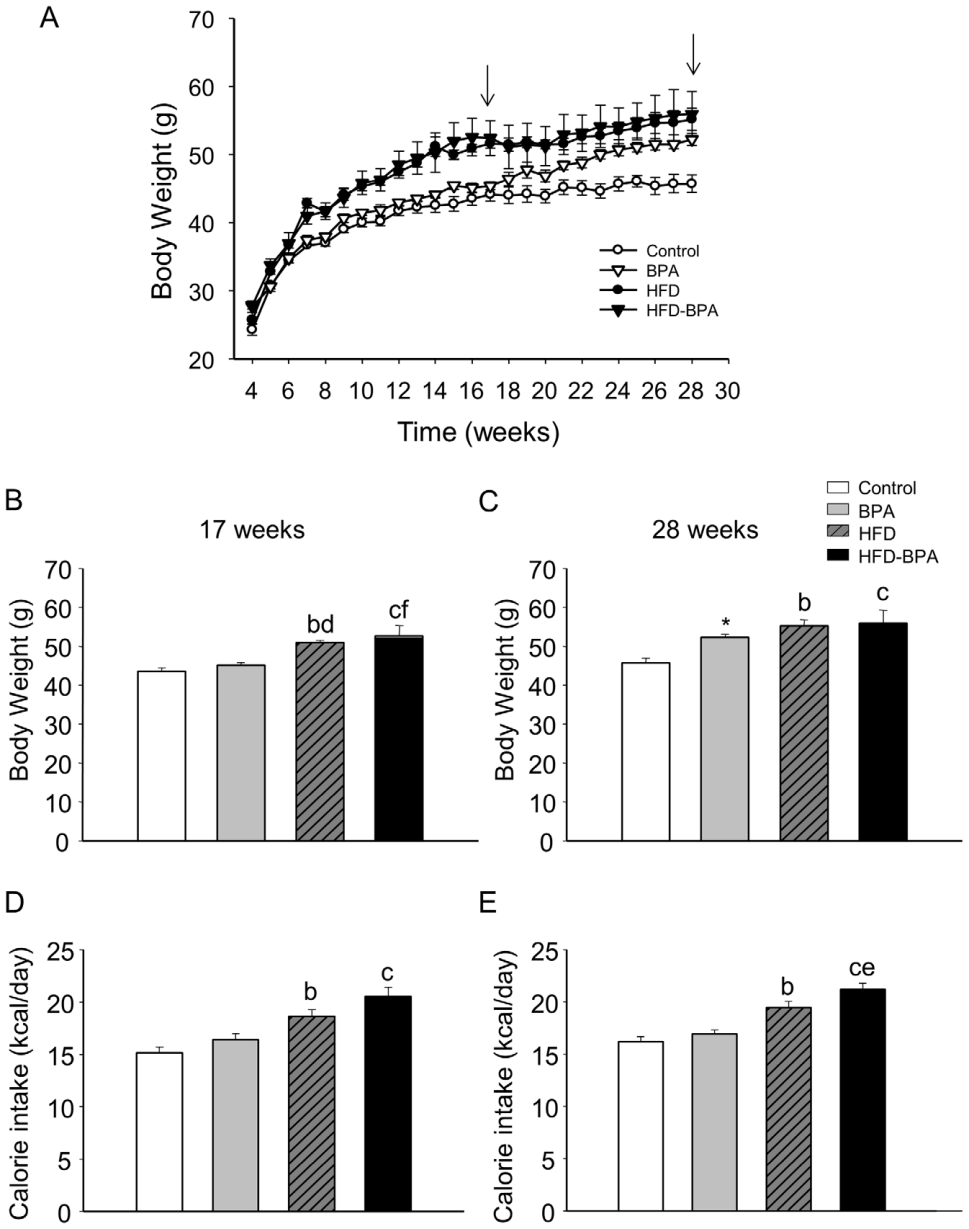
Body weight and calorie intake in the four study groups. **A**) Body weight evolution from 4 weeks to 28 weeks old. Animals were started on the HFD at 4 weeks. Arrows indicate weeks 17 and 28, the age when experiments were done (n = 150 animals from 26 litters). **B**) Body weight of offspring from the four different groups at 17 weeks (Control n = 46 animals from 8 litters, BPA n = 28 from 5 litters, HFD n = 38 from 8 litters and HFD-BPA n = 30 from 5 litters). **C**) Body weight of offspring from the four different groups at 28 weeks (Control n = 19 animals from 8 litters, BPA n = 15 from 5 litters, HFD n = 26 from 8 litters and HFD-BPA n = 15 from 5 litters), *P<0.01 Student's t-test compared to Control. **D**) Food intake for the four different groups compared at 17 weeks old (n = 142 animals from 26 litters) **E**) Food intake for the four different groups compared at 28 weeks old (n = 75 animals from 26 litters). Data are expressed as mean±SEM. Significance by one way ANOVA followed by Tukey, Holm-sidak or Dann's method, p<0.05. b compares control vs. HFD, c control vs. HFD-BPA, d HFD vs. BPA, e HFD vs. HFD-BPA, f BPA vs. HFD-BPA.

Body weights (BWs) at birth was diminished in BPA-treated mice (data not shown). BWs from the different groups were measured weekly starting at the age of 4 weeks (Figure 1A). HFD and HFD-BPA groups presented a higher weight than those fed a ND during the whole treatment. Remarkably, those in the BPA group started to gain more weight in the 18th week compared with the Control and from that point on their weight increased until it became similar to that of the HFD and HFD-BPA groups (figure 1A). The weight increase of the BPA group was not significantly different when compared with the rest of the groups using ANOVA either at the age of 17 weeks or 28 weeks (Figure 1B and C). However, at the age of 28 weeks, it was significantly different when compared with the Control as shown by the use of student's t-test (Figure 1C). Calorie intake remained unchanged in BPA mice compared with the Control group throughout the study (Figure 1D and E), yet it was increased in HFD and HFD-BPA groups compared with Control (Figure 1D and E) and remarkably, in HFD-BPA compared with HFD when they were 28 weeks old (Figure 1E).

**Figure 2:**
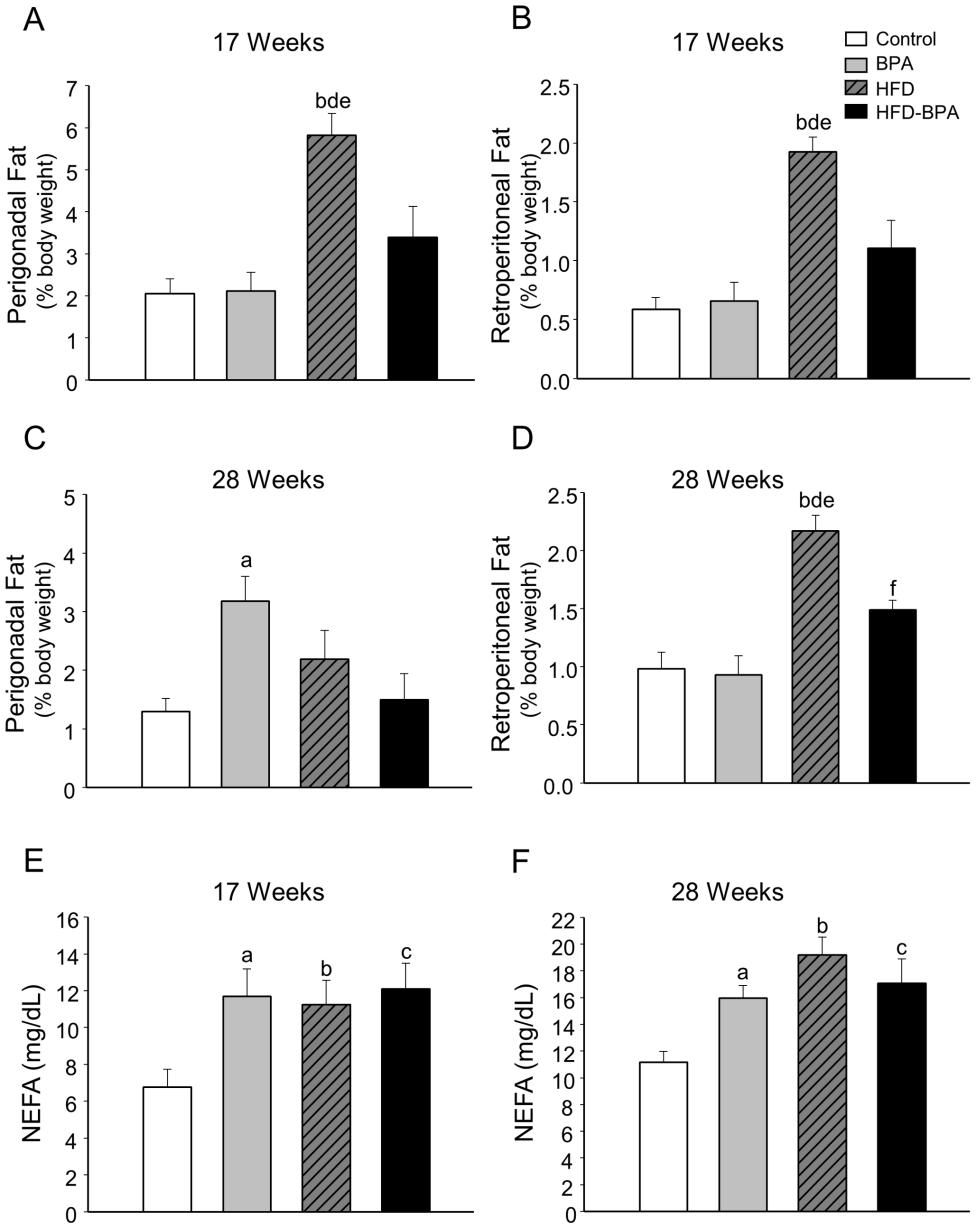
Adipose tissue weight and plasma NEFA levels in offspring. **A**) Perigonadal and **B**) Retroperitoneal fat depots, expressed as % of body weight at 17 weeks old (n = 8 animals from 5–8 litters). **C**) Perigonadal and **D**) Retroperitoneal fat depots, expressed as % of body weight at 28 weeks old. (n = 6–8 animals from 5–8 litters). **E**) Plasma Nonesterified fatty acids (NEFAs) levels at 17 weeks old (n = 7–9 animals from 7 litters) and **F**) 28 weeks old (n≥5 animals from 4–7 litters). Data are expressed in mean ±SEM; significance p<0.05 by one way ANOVA followed by Holm-Sidak or Dunnett's method; a control vs. BPA; b control vs. HFD, c control vs. HFD-BPA, d HFD vs. BPA, e HFD vs. HFD-BPA, f BPA vs. HFD-BPA.

By the age of 17 weeks the HFD animals were observed to have significant differences in both the perigonadal and retroperitoneal fat pad weights compared with the rest of the groups. It should be noted that the HFD-BPA group presented a diminished perigonadal and retroperitoneal fat pad weight compared with the HFD one (Figure 2A and B). Later, at the age of 28 weeks, perigonadal fat pad weight was significantly increased in the BPA group compared with the Control (Figure 2C). As for the retroperitoneal fat pad; its weight was increased in the HFD group compared with the rest of the groups and despite being higher in the HFD-BPA group than in the Control one, it was significantly lower compared with the HFD group (Figure 2D). No significant differences were observed with BPA treatment in the weight of the liver and gastrocnemius muscle (Figure S1).

Plasma non esterified fatty acid (NEFA) levels were increased in all groups, BPA, HFD and HFD-BPA compared with the control group at 17 and 28 weeks old (Figure 2E and F).

### Glucose tolerance and insulin sensitivity

**Figure 3:**
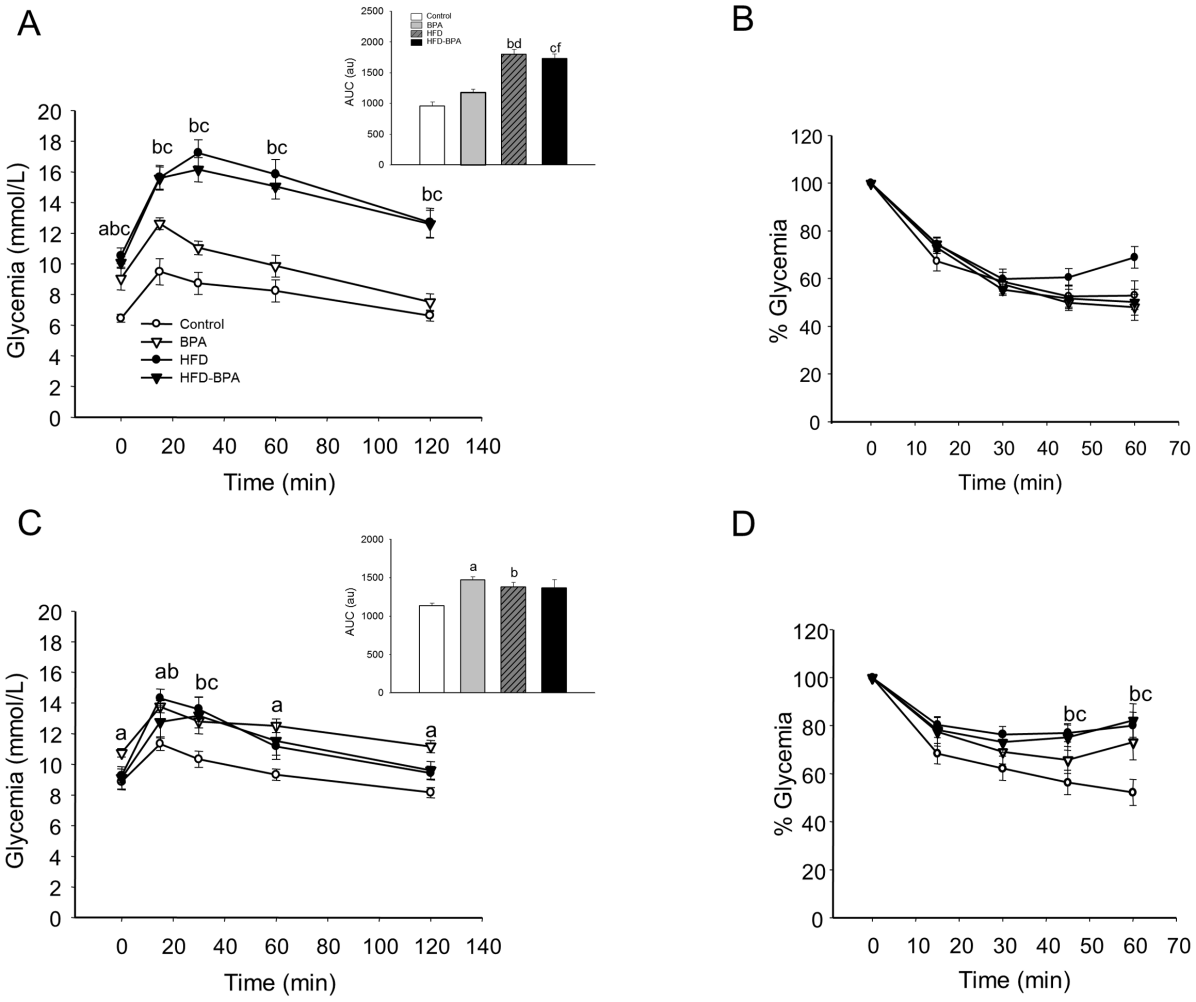
BPA, HFD and HFD-BPA groups exhibit hyperglycaemia and glucose intolerance. **A**) ipGTT were performed on the four groups at 17 weeks old. The inset shows mean±SEM of the ipGTT Area Under the Curve (n≥6 animals from ≥6 litters). **B**) ipITT in 17 week old animals (n≥6 animals from ≥6 litters) **C**) ipGTT were performed on the four groups at 28 weeks old. The inset shows mean±SEM of the ipGTT Area Under the Curve (n≥12 animals from ≥6 litters). **D**) ipITT in 28 week old animal. (n≥11 animals from ≥6 litters). Data are expressed in mean±SEM. Significance P<0.05 by one way ANOVA followed by Tukey or Dunnett's method; a control vs. BPA; b control vs. HFD, c control vs. HFD-BPA, d HFD vs. BPA, e HFD vs. HFD-BPA, f BPA vs. HFD-BPA.

In 17-week-old animals, a difference in fasting blood glucose levels was observed between the control group and the rest of the groups (table 1 and figure 3A). Remarkably, the BPA group was hyperglycemic in the fasted state compared with the control while no significant differences were found between the BPA, HFD and HFD-BPA groups (Table 1 and Figure 3A). In the fed state, no significant differences were found in glucose levels and plasma insulin between the 4 different groups using ANOVA one way (Table 1). The BPA, HFD and HFD-BPA groups presented higher insulin levels compared with the control one when each condition was compared with the control using student's t-test (p<0.05) and presented a high tendency when Kruskal-Wallis one way ANOVA on ranks was used (p<0.1). In 17 week old mice, glucose tolerance was impaired in the HFD and HFD-BPA groups compared with the Control, while HFD and HFD-BPA presented no significant difference (Figure 3A, see inset). The BPA group showed a tendency to be glucose intolerant although no significant differences were found compared with the Control (Figure 3A, see inset). When we used an intraperitoneal insulin tolerance test (ipITT) to study insulin sensitivity, no change was obtained between the 4 different groups (Figure 3B).

**Table 1:** Plasma hormones and metabolite levels in offspring at the age of 17 weeks.

	17 Weeks			
	Control	BPA	HFD	HFD+BPA
**Glucose Fasted State (mg/dL)**	121±6	163±13^a^	190±9^b^	182±7^c^
**Glucose (mg/dL)**	189±8	209±13	210±14	218±6
**Insulin (ng/mL)**	2.0±0.3	3.9±0.6	4.0±0.9	3.4±0.5
**TG (mg/mL)**	0.98±0.1	1.19±0.3	1.12±0.1	0.98±0.1
**Glycerol (mg/mL)**	0.20±0.04	0.23±0.03	0.35±0.04^b^	0.28±0.05

All measurements were performed in the fed state, otherwise stated. Fasting glucose was measured after 6 hours of fasting (n≥6 animals from ≥5 litters). Data are expressed in mean±SEM. Significance p<0.05 by one way ANOVA followed by Dunnet's method;

a control vs. BPA;

b control vs. HFD,

c control vs. HFD-BPA.

*p<0.05 Students t-test, comparing each condition with Control and p<0.1 using Kruskal-Wallis one way ANOVA on ranks.

In 28-week-old animals, the BPA group presented hyperglycaemia in contrast to the control group (Table 2 and Figure 3C). Moreover, the AUC of the BPA and HFD groups were significantly different compared with the control one but presented no significant difference when compared with each other (figure 3C, inset). In the fed state, no statistically significant difference was observed in plasma glucose and insulin levels (table 2), although a high tendency towards hyperinsulinaemia was seen in the BPA group. Insulin sensitivity had a tendency to be impaired in all groups compared with the Control one, yet no significant differences were obtained during the first 45 minutes after injecting insulin (Figure 3D). When comparing just Control and HFD conditions we found statistically significant differences in the glycemia levels in response to insulin (Figure S3).

**Table 2:** Plasma hormones and metabolite levels in 28 week old offspring.

	28 Weeks			
	Control	BPA	HFD	HFD+BPA
**Glucose Fasted State (mg/dL)**	163±7	193±5^a^	167±8	155±12^f^
**Glucose (mg/dL)**	186±4	209±7	180±7^d^	183±12
**Insulin (ng/mL)**	2.6±0.4	3.9±1.3	2.5±0.8	2.1±0.7
**TG (mg/mL)**	1.5±0.2	1.93±0.2	1.3±0.2	1.01±0.1
**Glycerol (mg/mL)**	0.36±0.04	0.39±0.03	0.38±0.03	0.36±0.05

All measurements were performed in the fed state, otherwise stated. Fasting glucose was measured after 6 hours of fasting. (n≥5 animals from ≥5 litters). Data are expressed in mean±SEM. Significance p<0.05 by one way ANOVA followed by Dunnet's method;

a control vs. BPA;

d HFD vs. BPA,

f BPA vs. HFD-BPA.

The change induced by HFD on glucose tolerance seems to be more pronounced at 17 than at 28 weeks of age, we believe this could be related to a deterioration of glucose tolerance in vehicle treated mice with age.

In summary, these experiments pointed to the fact that animals treated with BPA had an impaired glucose tolerance when compared with the Control. Remarkably, the phenotype of the BPA group resembles that of HFD mice.

### The effect of BPA on glucose stimulated insulin secretion (GSIS) and insulin content

**Figure 4:**
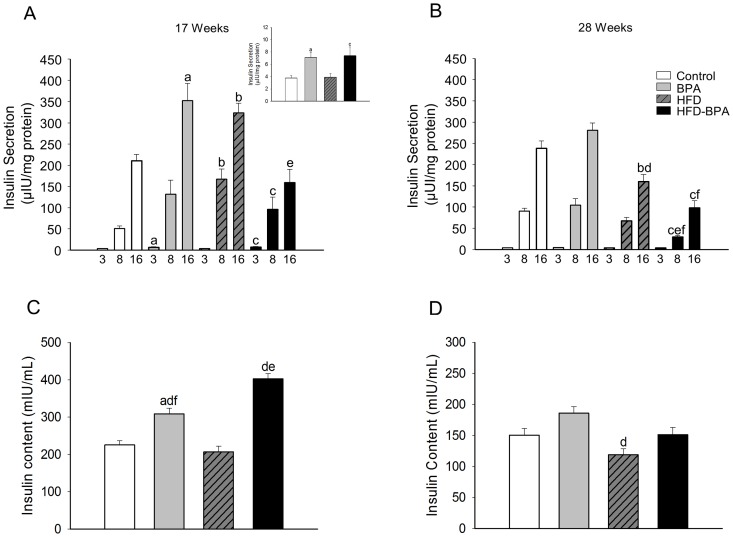
Glucose-induced insulin secretion from isolated islets. **A**) Insulin secretion from islets exposed to 3, 8 and 16 mM glucose for 1 hour, in animals from the four different groups at the age of 17 weeks. (n≥9 experiments with 7 animals from 7 litters). The inset shows the insulin release when islets were exposed to 3 mM of glucose. **B**) Insulin secretion from islets exposed to 3, 8 and 16 mM of glucose for 1 hour, in animals from the four different groups at the age of 28 weeks (n≥6 experiments with ≥6 animals from ≥6 litters). **C**) Insulin content from isolated islets at the age of 17 weeks (n≥10 experiments with 7 animals from 7 litters). **D**) Insulin content from isolated islets at 28 weeks (n≥8 experiments with ≥6 animals from ≥6 litters). Data are expressed in mean±SEM, p<0.05 by one way ANOVA followed by Tukey, Dunnet or Dunn's method; a control vs. BPA; b control vs. HFD, c control vs. HFD-BPA, d HFD vs. BPA, e HFD vs. HFD-BPA, f BPA vs. HFD-BPA.

Islets from adult offspring whose mothers were treated with BPA during pregnancy, feeding with either HFD or ND, presented an increased insulin secretion in response to basal levels of glucose, 3 mM, (Figure 4A inset) as well as in response to stimulatory glucose levels, 16 mM at 17 weeks old (Figure 4A). HFD treated animals presented an increase of GSIS similar to that found in the BPA group, even though basal insulin secretion at 3 mM of glucose was not increased. The HFD-BPA group showed an enhanced insulin release in response to 3 and 8 mM of glucose when compared to the control but a diminished GSIS in response to 16 mM of glucose when compared with the BPA group, which suggests that BPA exposure may have a detrimental action on high glucose stimulated insulin secretion when combined with a HFD.

No difference in insulin secretion between the BPA and Control groups was obtained in 28-week-old animals, yet the HFD and HFD-BPA groups showed a significantly diminished GSIS when compared with the control and BPA groups. Notably, insulin release in response to 8 and 16 mM of glucose was decreased in the HFD-BPA group compared with the HFD one (Figure 4B). This result indicates that GSIS was progressively worsened by HFD in offspring whose mothers were exposed to BPA during pregnancy and therefore, the effect of HFD was exacerbated by intra uterine BPA exposure (Figure 4B).

At the age of 17 weeks, islet insulin content was significantly higher in the BPA and HFD-BPA groups compared with the Control one, however, the Control and HFD groups did not present differences (Figure 4C). At the age of 28 weeks, the situation was different. There were no significant differences between the Control, BPA and HFD-BPA groups and the HFD group presented an insulin content slightly diminished compared with the Control (Figure 4D).

In order to evaluate if the stimulus-secretion pathway was altered, glucose induced Ca^2+^ signaling was monitored. In 17 week old animals, [Ca^2+^]_i_ oscillations induced by 8 and 16 mM of glucose were unaltered in all the measured conditions (Figure S1 A–C). In 28 week old animals fed a ND, the BPA group presented a higher [Ca^2+^]_i_ response to 16 mM of glucose (Figure S2) which was blunted in HFD animals.

### The effects of BPA on gene expression in white adipose tissue, liver and skeletal muscle

White adipose tissue (WAT), the liver and skeletal muscle are key organs involved in the metabolism of lipids and carbohydrates. We measured the changes in the expression of several metabolic genes involved in lipogenesis, fatty acid uptake, fatty acid oxidation and glucose uptake and metabolism at the age of 17 weeks. This age was chosen because it was just prior to the moment when weight increases. Therefore, the observed changes may have been involved in the process of weight increase.

**Figure 5:**
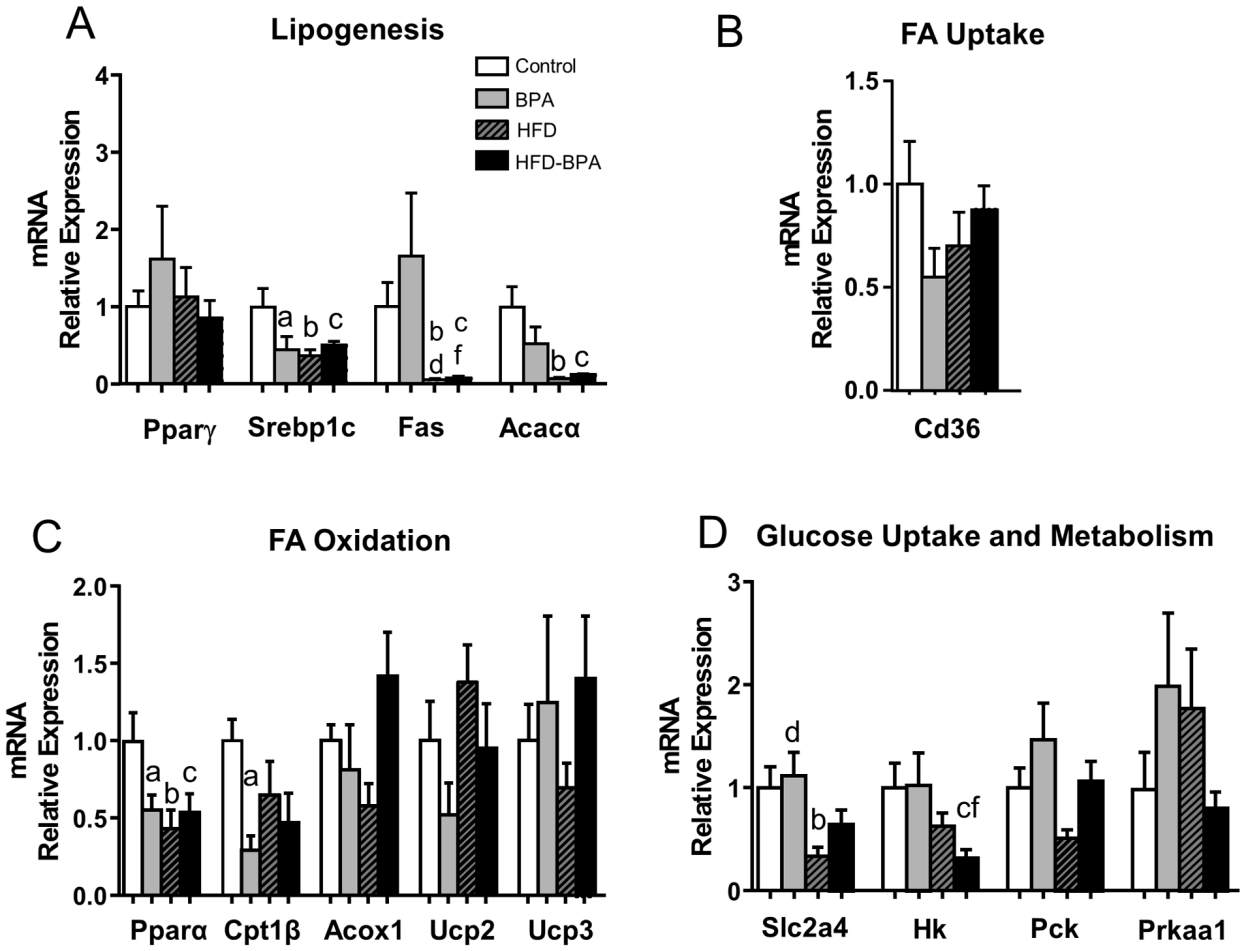
mRNA gene expression associated with A) Lipogenesis, B) Fatty Acid Uptake, C) Fatty Acid Oxidation and D) Glucose Uptake and Metabolism, in White Adipose Tissue (WAT) from Control (n≥5 animals from ≥5 litters), BPA (n≥5 animals from ≥5 litters), HFD (n≥5animals from ≥5 litters) and HFD-BPA (n≥5 animals from ≥5 litters) dams at 17 weeks old. Animals were treated with HFD at week 4. Gene expression was assessed by real-time RT-PCR. Data are expressed as mean±SEM and statistical significance was determined using one way ANOVA followed by Tukey, Duncans or Dunn's method. Significance p<0.05; a compared control vs. BPA; b control vs. HFD, c control vs. HFD-BPA, d HFD vs. BPA, e HFD vs. HFD-BPA, f BPA vs. HFD-BPA.

In WAT (perigonadal pad), a significant decrease in *Srebp1c* (Figure 5A), *Pparα* and *Cpt1β* (Figure 5C) mRNA levels was found in the BPA group compared with the Control. In the case of *Srebp1c* and *Pparα*, the changes in BPA were equal to those found in HFD. *Acacα* and *Cd36* expression tended to decrease (Figure 5A and B) although the results were not statistically significant. Fas expression was suppressed in the HFD and HFD-BPA groups to the same extent, which was expected as a consequence of the decrease in *Srebpc1* expression. Remarkably, *Fas* mRNA expression was not modified in the BPA group compared with the Control (Figure 5A). Hexokinase mRNA expression was markedly down regulated by HFD-BPA compared to Control (Figure 5D).

**Figure 6:**
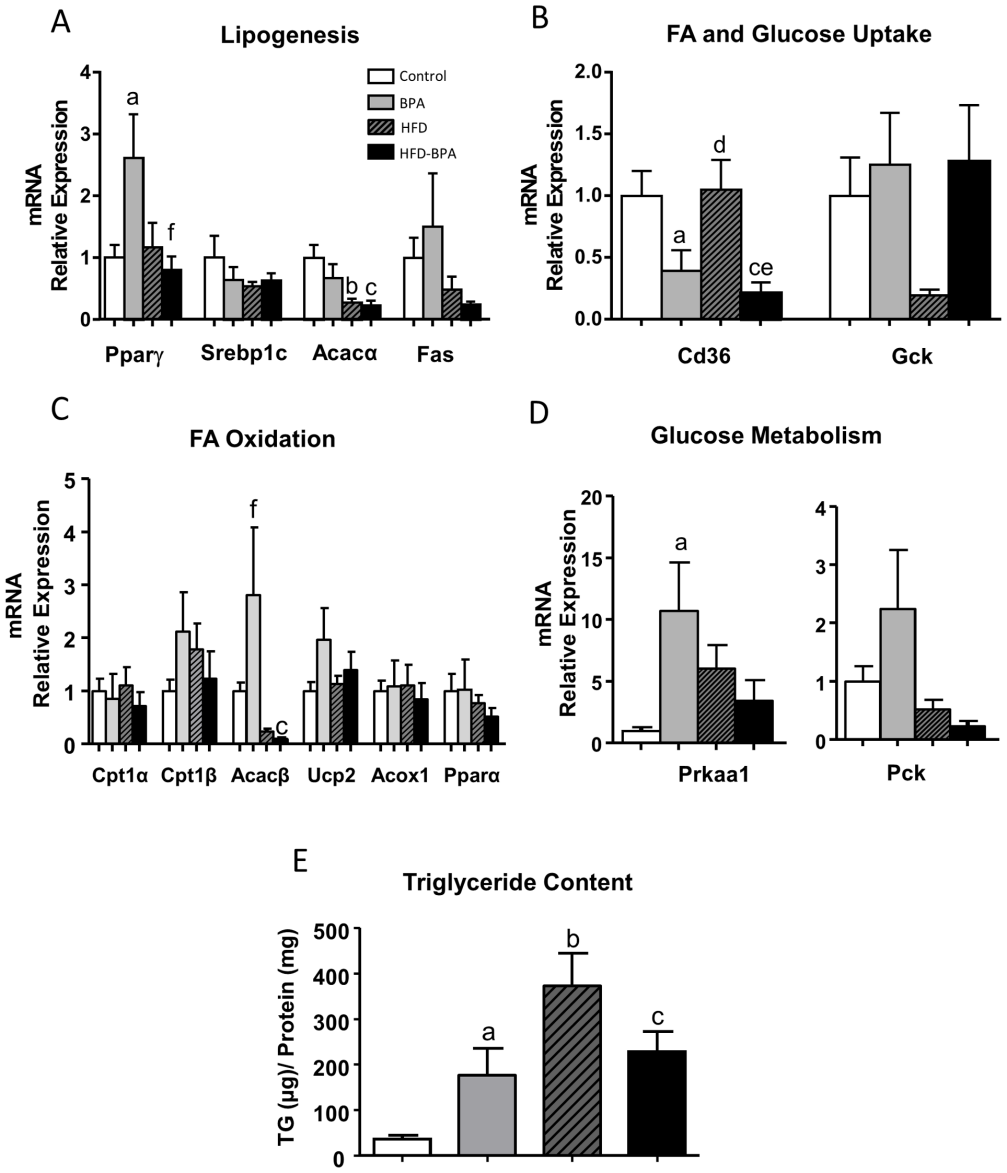
mRNA gene expression associated with A) Lipogenesis, B) Fatty Acid and Glucose Uptake, C) Fatty Acid Oxidation and D) Glucose Metabolism, in the liver from Control (n≥5animals from ≥5 litters), BPA (n≥5 animals from ≥5 litters), HFD (n≥5 animals from ≥5 litters) and HFD-BPA (n≥5 animals from ≥5 litters) dams at the age of 17 weeks. Animals started to be treated with HFD at the age of 4 weeks. Gene expression was assessed by real-time RT-PCR. E) Hepatic trygliceride content from Control (n≥5animals from ≥5 litters), BPA (n≥5 animals from ≥5 litters), HFD (n≥5 animals from ≥5 litters) and HFD-BPA (n≥5 animals from ≥5 litters) dams at the age of 17 weeks. Data are expressed as mean±SEM and statistical significance was determined using one way ANOVA followed by Tukey or Dunn's method. Significance p<0.05; a compares control vs. BPA; b control vs. HFD, c control vs. HFD-BPA, d HFD vs. BPA, e HFD vs. HFD-BPA, f BPA vs. HFD-BPA.

In the liver, mRNA levels of *Pparγ* were increased in BPA treated mice, which differed from what was observed in the HFD and HFD-BPA groups that remained the same as the Control (Figure 6A). The mRNA expression of *Cd36* involved in FA uptake was significantly decreased in the BPA and HFD-BPA groups compared with the Control and HFD (Figure 6B). *Acacβ* levels were highly increased in BPA group; an opposite effect to the one produced in the HFD and HFD-BPA groups (Figure 6C) yet the difference was not significant compared with the control. The relative expression of *Prkaa*, the AMPK gene, was significantly increased in the BPA group and showed a tendency to increase in the HFD and HFD-BPA groups (Figure 6D). *Pparα* and *Pepck* expression, involved in fatty acid oxidation and gluconeogenesis was not significantly changed by any of the treatments (Figure 6C, D). In view of these results we measured the hepatic levels of triglycerides. We found an increase in all groups of animals compared to control one. This increase was more pronounced in the HFD group but we also found a significant increment in the BPA group (Figure 6E).

**Figure 7:**
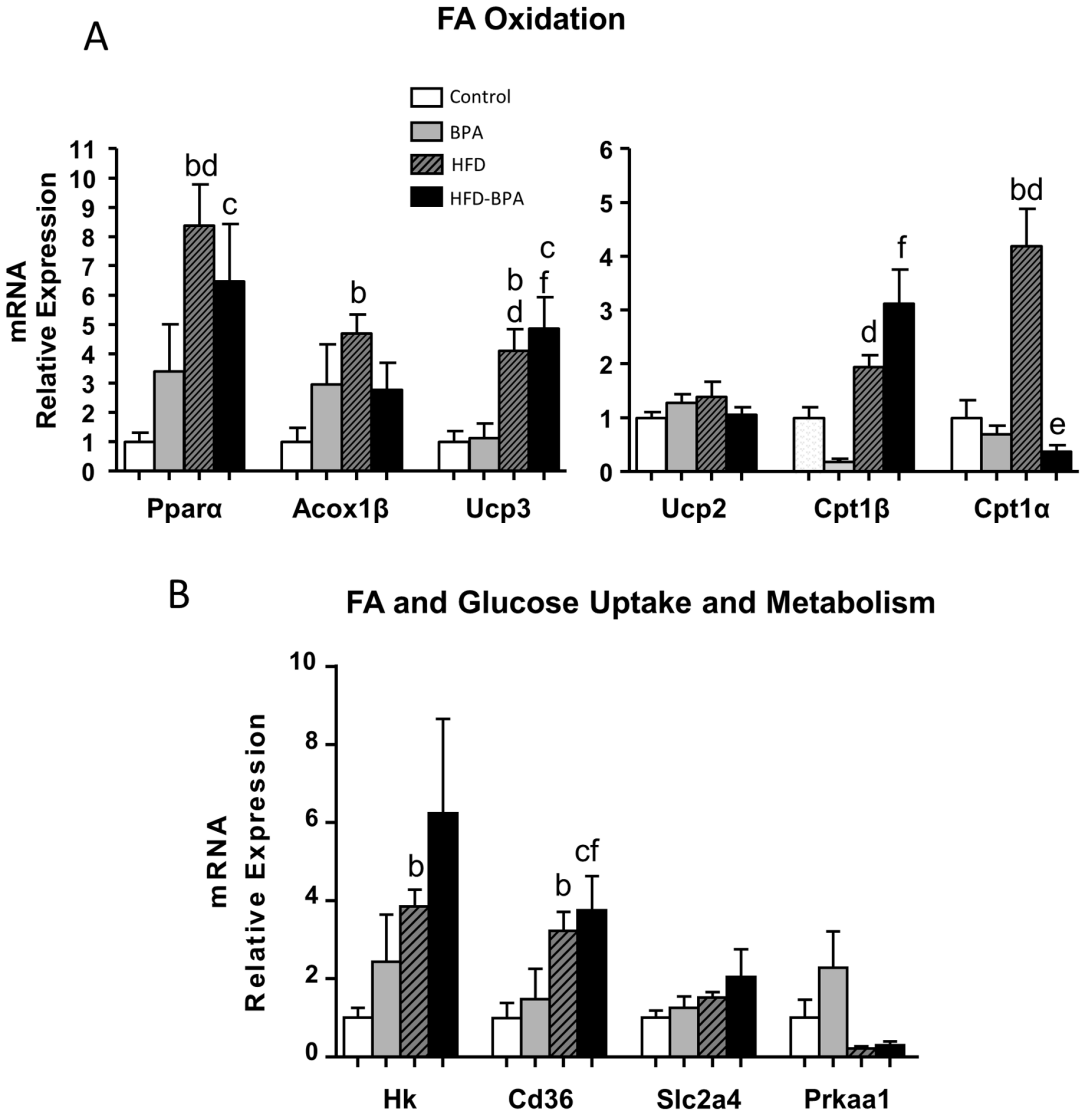
mRNA gene expression associated with A) Fatty Acid Oxidation and B) Fatty Acid and Glucose Uptake and Metabolism, in Skeletal Muscle from Control (n≥5 animals from ≥5 litters), BPA (n≥5 animals from ≥5 litters), HFD (n≥5 animals from ≥5 litters) and HFD-BPA (n≥5 animals from ≥5 litters) dams at the age of 17 weeks. Animals started to be treated with HFD at week 4. Gene expression was assessed by real-time RT-PCR. Data are expressed as mean±SEM and statistical significance was determined using one way ANOVA followed by Duncan or Dunn's method. Significance p<0.05; a compared control vs. BPA; b control vs. HFD, c control vs. HFD-BPA, d HFD vs. BPA, e HFD vs. HFD-BPA, f BPA vs. HFD-BPA.

In skeletal muscle (gastrocnemius), the mRNA levels of the transcription factor involved in fatty acid oxidation, *Pparα* and *Acox1β* were markedly up regulated by HFD. A tendency to increase was present in the BPA and HFD-BPA groups although the difference was not statistically significant. Remarkably, the increase in *Cpt1α* induced by HFD was completely abrogated in the HFD-BPA group (Figure 7A). The levels of hexokinase as well as those of *Cd36* increased significantly only in the HFD group (Figure 7B). No significant changes were observed in *Glut 4* and AMPK (Figure 7B).

## Discussion

The exposure to endocrine disrupters has been unveiled as a risk factor for type 2 diabetes mellitus. Numerous evidence exists in animal and epidemiological studies to establish a link between EDC exposure and T2D [10], [30], [31], [32]. These endocrine disruptors include persistent organic pollutants, heavy metals and plastic components such as phthalates and bisphenol-A, among others that are presently emerging. In addition, other EDCs have been described as obesogenic, such as tributyltin, triflumizole [33], [34] and DES [35], [36].

The present study together with previous observations [20], [23], [24], [37] have demonstrated that the exposure to low doses of BPA during pregnancy alters glucose homeostasis in male offspring. We have demonstrated here that glucose homeostasis and mRNA expression of genes involved in the glucose and lipid metabolism in WAT, liver and skeletal muscle are altered by prenatal exposure to BPA in a similar manner to that of HFD fed animals unexposed to BPA. Moreover, some important HFD effects were worsened by BPA exposure.

The body weight of BPA animals reached levels comparable to those of HFD at 20 weeks. This was unexpected, because previous work from our group had not registered an increase in weight using the same treatment (days 9–16 of pregnancy) [23]. The reason for this difference is unknown. It is possible that the use of different animals (from the same strain) or small unpredicted differences in the batches of diet food may have had an influence on the results. It is of note that at week 17, 2 mice/cage were removed, so the cage density changed from 8mice/group to 6 mice/group. This is, however, unlikely to affect weight only in the BPA-treated group, because this change was performed in all groups including control. In a recent article, a non-monotonic relationship between BPA exposure during pregnancy and weight increase in offspring was reported [24]. Only those animals whose mothers were exposed to BPA 500 µg/kg/day presented a significant higher weight at the age of 18 weeks. It is plausible that the difference in the levels of BPA to which fetuses are exposed may change from one experiment to another and might be responsible for the variations in weight increase. In any case, new experiments are required to unequivocally solve the question of whether exposure during pregnancy is critical to elicit weight increase later in life. There is more evidence; however, to suggest that perinatal exposure (during pregnancy and lactation) to BPA, increases body weight in mice [19], [38], [39] and rats [20], [40]. Therefore, BPA exposure during the lactation period may be critical to induce obesity later in life [41]. In humans, evidence has been recently published suggesting an association between urinary BPA levels in children and obesity [42], yet more studies are needed to establish a solid link.

Perigonadal fat pad weight increased with BPA at 28 weeks. Surprisingly in the HFD-BPA group the weight of the perigonadal fat pad, was decreased at 17 and 28 weeks without a change in the total body weight. Notably, perigonadal fat mass was increased in the BPA group, a phenomenon that will be explored in the future. In all cases, NEFA levels were highly increased at both ages when compared with the Control. High NEFA levels in plasma are a risk factor for metabolic syndrome and induce β-cell death [43], [44], [45], [46]. Fasted glucose levels were also increased to the same extent at 17 weeks in the BPA, HFD and HFD-BPA groups. High glucose levels together with high levels of NEFAs, could be partly responsible not only for the increase in glucose stimulated insulin secretion (GSIS) in the BPA and HFD groups at 17 weeks as a compensatory response, but for the subsequent decrease of GSIS at 28 weeks because of glucolipotoxicity. Interestingly, this decrease was clearly potentiated by BPA in the HFD-BPA group, indicating that BPA may exacerbate the effect of HFD on pancreatic β-cell function. These different processes have been described when the compensatory adaptive state progresses to diabetes in obese individuals [44], [47], [48], [49], [50].

BPA exposure during pregnancy predispossed offspring to develop glucose intolerance when a glucose tolerance test was performed after 6 h fasting. At 17 weeks of age the group of animals that have been treated with HFD are already glucose intolerance, meanwhile BPA showed a clear tendency to that. At 28 weeks of age, BPA mice presented glucose intolerance to the same extent that HFD group. No difference was obtained between the HFD and HFD-BPA groups, unlike what was described in rats in which perinatal exposure to BPA exacerbated the action of HFD in male offspring [20].

No differences in insulin sensitivity among the groups were reported, a slightly different result to the one described in Alonso-Magdalena et al [23]. In that report, animals exposed to 10 µg/kg/day presented glucose intolerance and insulin resistance while those exposed to 100 µg/kg/day were glucose intolerant with no change in insulin sensitivity. These results suggested that BPA exposure and insulin sensitivity could be related in a non-monotonic dose dependent manner. Recently, Angle et al [24], have demonstrated the existence of this non-monotonic relationship using a full range of BPA doses. Insulin sensitivity decreased at 5 and 5000 µg/kg/day of BPA but remained normal at 50, 500 and 50000 µg/kg/day of BPA. Non-monotonic dose responses are a general phenomenon that occurs with some EDCs, including BPA [51]. Therefore, fetuses may be exposed to different BPA levels in blood depending on the mother's BPA metabolism, on whether BPA is administered: by injection, gavage or using a micropipetter among other variables. Small variations in the real dose of BPA may exist between one experiment and another. This might be responsible for the differences in insulin sensitivity, weight changes and other endpoints found in different reports. It is worth noting, however, that in most of the published studies, pregnancy and perinatal BPA exposure worsened glucose tolerance at any of the concentrations assayed from 5 and 50000 µg/kg/day [20], [23], [24], [37], [52]. Exposure to lower levels 2.5 ng/kg/day produced no effect on glucose tolerance [53].

When mRNA expression of key genes in WAT, liver and skeletal muscle was studied, the most significant changes appeared in WAT and the liver. In WAT, BPA imitated HFD in decreasing the transcription factor Srebp1c, which is involved in the regulation of lipid biosynthesis in animal cells and plays an important role in the regulation of lipogenic enzyme gene expression [54]. The main lipogenic enzymes controlled by *Srebp1c* are acetyl-CoA carboxylase (*Acc*) and fatty acid synthase (*Fas*). Here the mRNAexpression levels of both enzymes were decreased due to HFD treatment but not because of the exposure to BPA. Thus, it seems that an alternative mechanism may exist to regulate Fas gene expression in the BPA group.

Peroxisome proliferator-activated receptor-α (*Pparα*) is a dietary lipid sensor whose activation results in hypolipidemic effects and mediates the expression of genes promoting fatty acid beta-oxidation. BPA exposure decreases *Pparα* expression in WAT to the same extent as HFD. A decrease in *Pparα* should result in a reduced capacity to metabolize long-chain fatty acids, an effect reported in *Pparα* KO mice, that contributes to both increased adipose tissue stores with aging and to the obese phenotype of these mice [55]. In the present study, the decrease in *Pparα* occurs simultaneously with a decrease in carnitine palmitoyltransferase1β (*Cpt1β*) mRNA expression, particularly in the BPA group. *Cpt1β* is involved in mitochondrial fatty acid uptake and catalyzes the rate-limiting step of fatty acid oxidation. The transcription of *Cpt1* is regulated by a number of factors, including peroxisome proliferator-activated receptor-α, glucocorticoids, and insulin [56]. Its decrease in WAT could result in a lower rate of fatty acids delivery to other peripheral tissues; mainly the liver. Additionally, it could contribute to an increase in weight. This would explain why BPA treatment during pregnancy induced a decreased expression in key genes such as *Srebpc1*, *Pparα* and *Cpt1β* in offspring to the same extent as HFD during 13 weeks.

In the liver, BPA specifically increases *Pparγ* and AMPK expression and decreases the expression of *Cd36*. *Pparγ* expression in the liver is much lower than in skeletal muscle and adipose tissue [57], [58] but it may have a pivotal role because it controls the expression of genes associated with lipid metabolism. An increase in *PPARγ* expression by BPA may increase hepatic fat accumulation [57]. Indeed we do observe an increase of triglycerides content in the liver of BPA animals compared to control group. The expression of *Cd36* was highly decreased by exposure to BPA, contrary to the lack of its effect in the HFD group. *Cd36* is a fatty acid transport protein whose increased expression in the liver contributes to dyslipidemia before the onset of type 2 diabetes [59]. In our study, *Cd36* expression decreased in the BPA and HFD-BPA groups. This might reduce fatty acid uptake and thereby contribute to the increase in NEFA levels in plasma as is the case in CD36 KO mice with higher plasma triglyceride and NEFA levels [60]. AMPK mRNA levels were elevated in the BPA group. AMPK is a fuel sensor protein that inhibits anabolic pathways and activates catabolic pathways in the liver. Over activity of hepatic AMPK has a net effect of increasing fat oxidation and decreasing the rate of glucose oxidation [61], [62]. Because of its favorable global metabolic effects when activated, the higher expression of AMPK found in the present study may be a compensating mechanism to counteract the hyperglycaemia detected in the BPA, HFD and HFD-BPA groups, which probably is due to an incomplete blockade of liver gluconeogenesis.

In the skeletal muscle, HFD provoked a downregulation of *Pparα* and *Acox1β* gene expression, yet BPA produced no significant effect by itself.

During the last decade, environmentally relevant concentrations of Bisphenol-A have been demonstrated to alter pancreatic beta cell function [6], [63], [64], [65] and liver metabolism [66], [67] and to induce insulin resistance and hyperinsulinaemia in adult mice [17], [18]. Perinatal or pregnancy exposure to BPA elicited weight increase and adipocity in offspring [20], [24], [38], [39], [40]; altered glucose tolerance [20], [23], [24], [37], [52] and insulin sensitivity [23], [24], [37]. Our research has demonstrated that exposure to an environmentally relevant dose of BPA during pregnancy, alters the expression of important genes involved in lipogenesis and fatty acid oxidation in adipocytes as well as fatty acid uptake in the liver. These alterations are associated with increased NEFA levels in plasma which are a risk factor for type 2 diabetes. In fact, animals exposed to BPA that were fed a HFD during 24 weeks had a deteriorated GSIS compared to HFD fed animals. Moreover, BPA treatment worsened the HFD effect on GSIS indicating that exposure to BPA exacerbates the deleterious action of HFD in the pancreatic β-cell. Phenotypically, mice treated with BPA presented higher body weight, fasted hyperglycaemia and altered glucose tolerance. These parameters were not aggravated by HFD. Therefore, these mice suffered primary symptoms of diabesity, a term used to refer to a form of diabetes which typically develops in later life and is associated with being obese. Whether human exposure to bisphenol-A is high enough to cause similar actions to those found in animals is still a matter of debate [68]. In any case, the dose of BPA used in the present study (10 µg/kg/day), is clearly below the referenced dose established by the EPA (50 µg/kg/day) [69]. As regards its potential translation to a reasonable exposition dose in humans, recent studies have suggested that the average adult is exposed to concentrations of BPA, in the range of 0.4–1.5 µg BPA/kg BW per day [70], [71]. However, after exhaustively reviewing all available human studies, scientists have reported that daily intake of BPA should be higher than initially thought and that, approximately 500 µg/kg/day would be required to account for the reported levels of BPA in adults [72]. Human exposure to BPA is variable but it has been measured in several studies and the central tendency is that unconjugated levels of BPA in humans are in the 0.3–4.4 ng/mL range in tissues and fluids in fetuses, children and adults; which exceed the circulating levels extrapolated from acute exposure studies in laboratory animals [73].

Above of that we must bear in mind that we are all exposed to a mixture of endocrine disruptors, including bisphenol-A, phthalates and persistent organic pollutants, which have been associated to type 2 diabetes and other metabolic alterations in human epidemiological studies. These EDCs may have additive effects in humans [74]. Although this possibility needs further exploration, it makes the exposure to EDC a real threat for metabolic disorders.

## Supporting Information

Figure S1 **Liver and gastrocnemius muscle weights.** **A**) Liver and **B**) gastrocnemius muscle weight, both expressed as % of body weight at the age of 17 weeks. (n≥6 animals from ≥5 litters). **C**) Liver and **D**) gastrocnemius muscle weight, both expressed as % of body weight at 28 weeks. (n≥8 animals from ≥5 litters). Data are expressed in mean ±SEM, *p<0.05 BPA vs all conditions by one way ANOVA followed by Dunnett's method.
(TIF)

Figure S2 **Calcium response to increased glucose concentration in isolated islets of animals exposed to BPA in uterus.** **A**) [Ca^2+^]_i_ response of a representative islet of Langerhans in response to 3, 8 and 16 mM glucose applied for 5, 11 and 11 min, respectively. **B**) Area under curve (AUC) was done during 10 min period in 8 mM glucose and **C**) in 16 mM of glucose in 17 week old offspring. **D**) Area under curve (AUC) during 10 min period in 8 mM of glucose and **E**) in 16 mM of glucose in 28 week old offspring. (n≥5 islets from ≥4 animals from ≥4 litters). Data are expressed in mean±SEM and analyzed by one way ANOVA followed by Holm-Sidak; *p<0.05.
(TIF)

Figure S3 **Insulin sensitivity is decreased in HFD animals compared to control at 28 weeks of age.** ipITT was performed in animals fed with HFD or chow diet during 24 weeks (n≥11 animals from ≥6 litters). Data are expressed in mean±SEM. Significance P<0.05 by t-test.
(TIF)

Table S1 **Real time PCR primers.**
(TIF)
