# Supplementary figures and images for: Exposure to Bisphenol-A during Pregnancy Partially Mimics the Effects of a High-Fat Diet Altering Glucose Homeostasis and Gene Expression in Adult Male Mice
Source: PLoS One. 2014 Jun 24;9(6):e100214. doi: 10.1371/journal.pone.0100214 (PMC4069068; doi:10.1371/journal.pone.0100214)

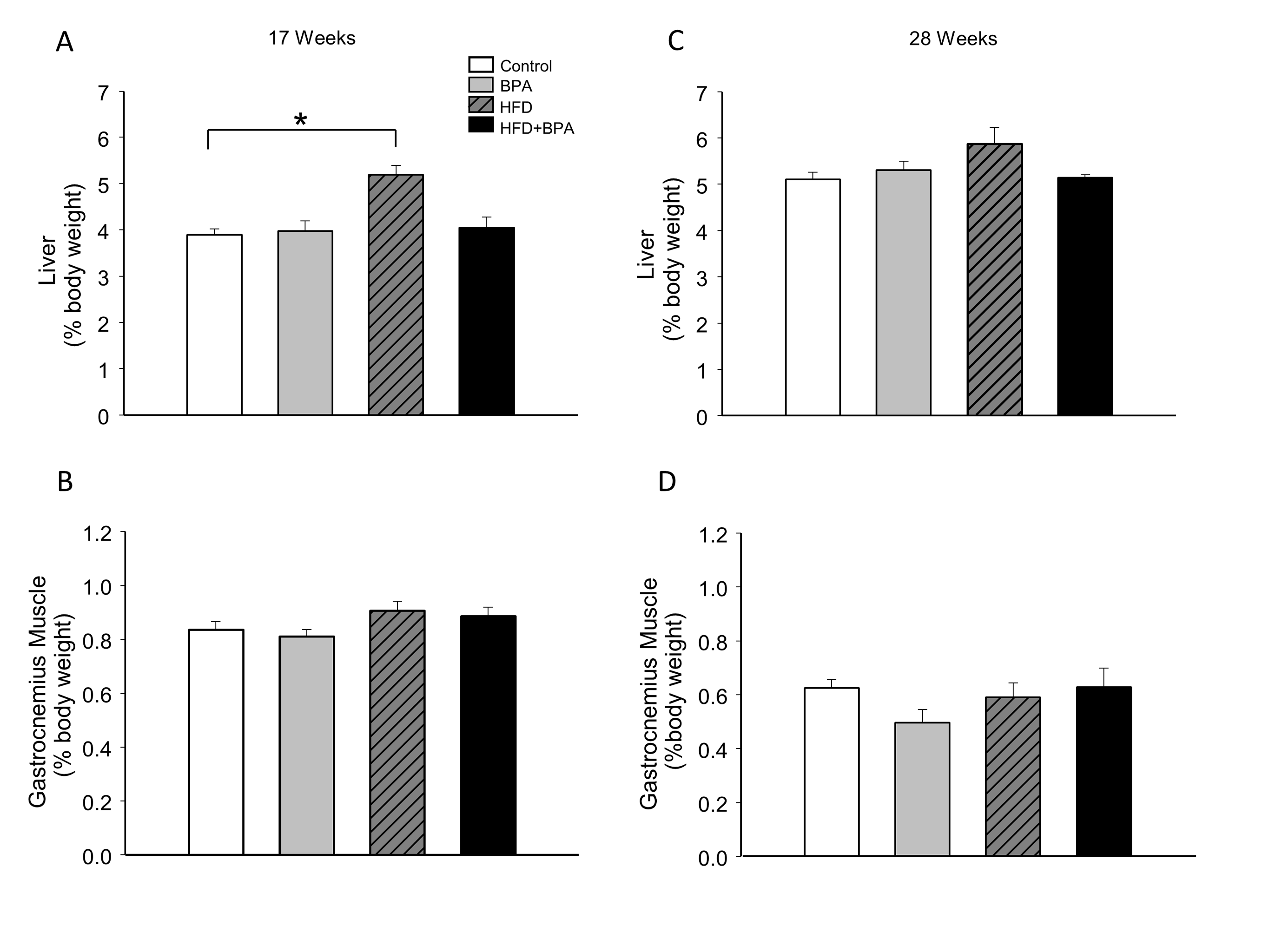

Supplement: Figure S1 — Liver and gastrocnemius muscle weights. A) Liver and B) gastrocnemius muscle weight, both expressed as % of body weight at the age of 17 weeks. (n≥6 animals from ≥5 litters). C) Liver and D) gastrocnemius muscle weight, both expressed as % of body weight at 28 weeks. (n≥8 animals from ≥5 litters). Data are expressed in mean ±SEM, *p<0.05 BPA vs all conditions by one way ANOVA followed by Dunnett's method. (TIF) [file pone.0100214.s001.tif]

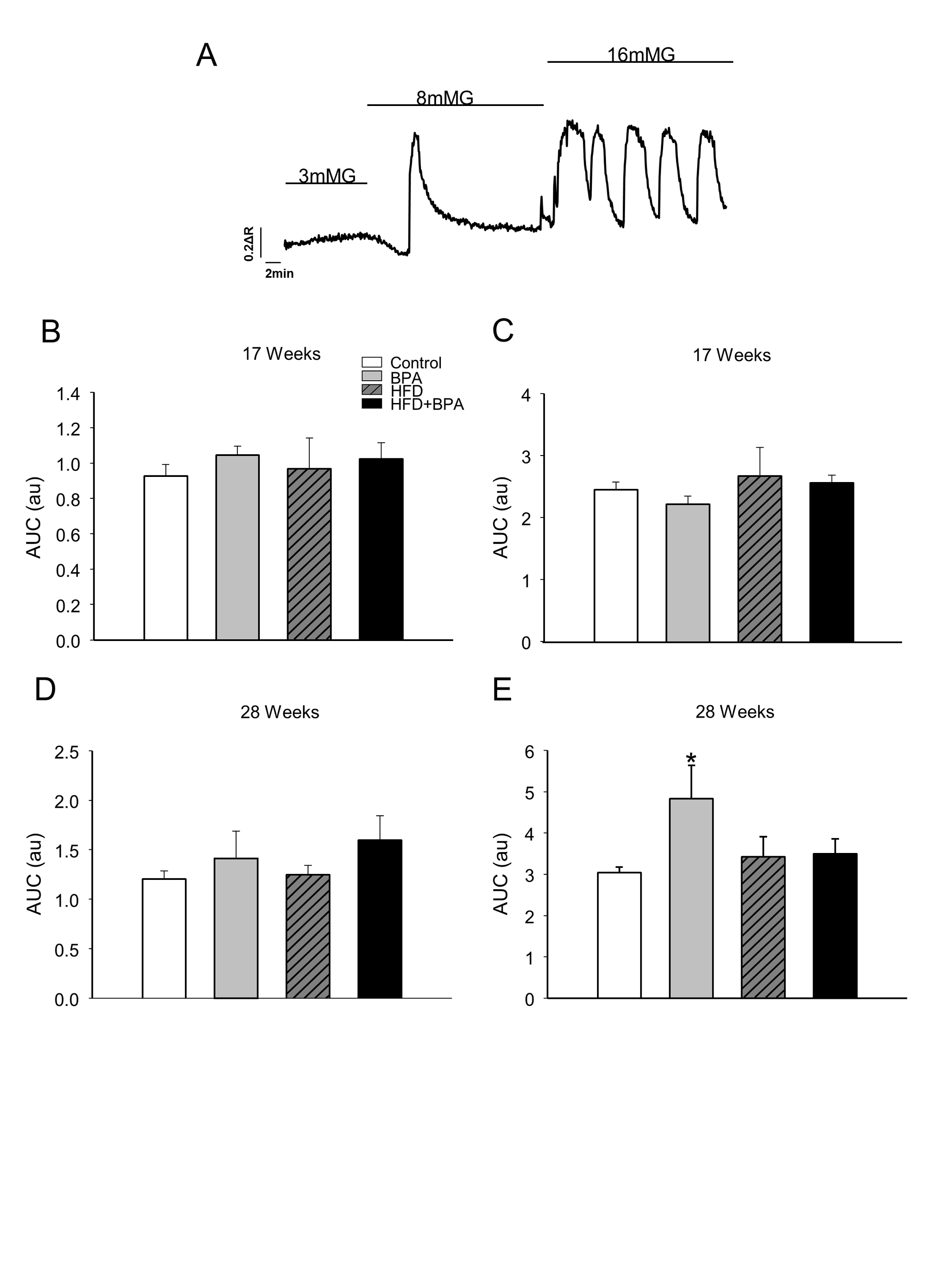

Supplement: Figure S2 — Calcium response to increased glucose concentration in isolated islets of animals exposed to BPA in uterus. A) [Ca2+]i response of a representative islet of Langerhans in response to 3, 8 and 16 mM glucose applied for 5, 11 and 11 min, respectively. B) Area under curve (AUC) was done during 10 min period in 8 mM glucose and C) in 16 mM of glucose in 17 week old offspring. D) Area under curve (AUC) during 10 min period in 8 mM of glucose and E) in 16 mM of glucose in 28 week old offspring. (n≥5 islets from ≥4 animals from ≥4 litters). Data are expressed in mean±SEM and analyzed by one way ANOVA followed by Holm-Sidak; *p<0.05. (TIF) [file pone.0100214.s002.tif]

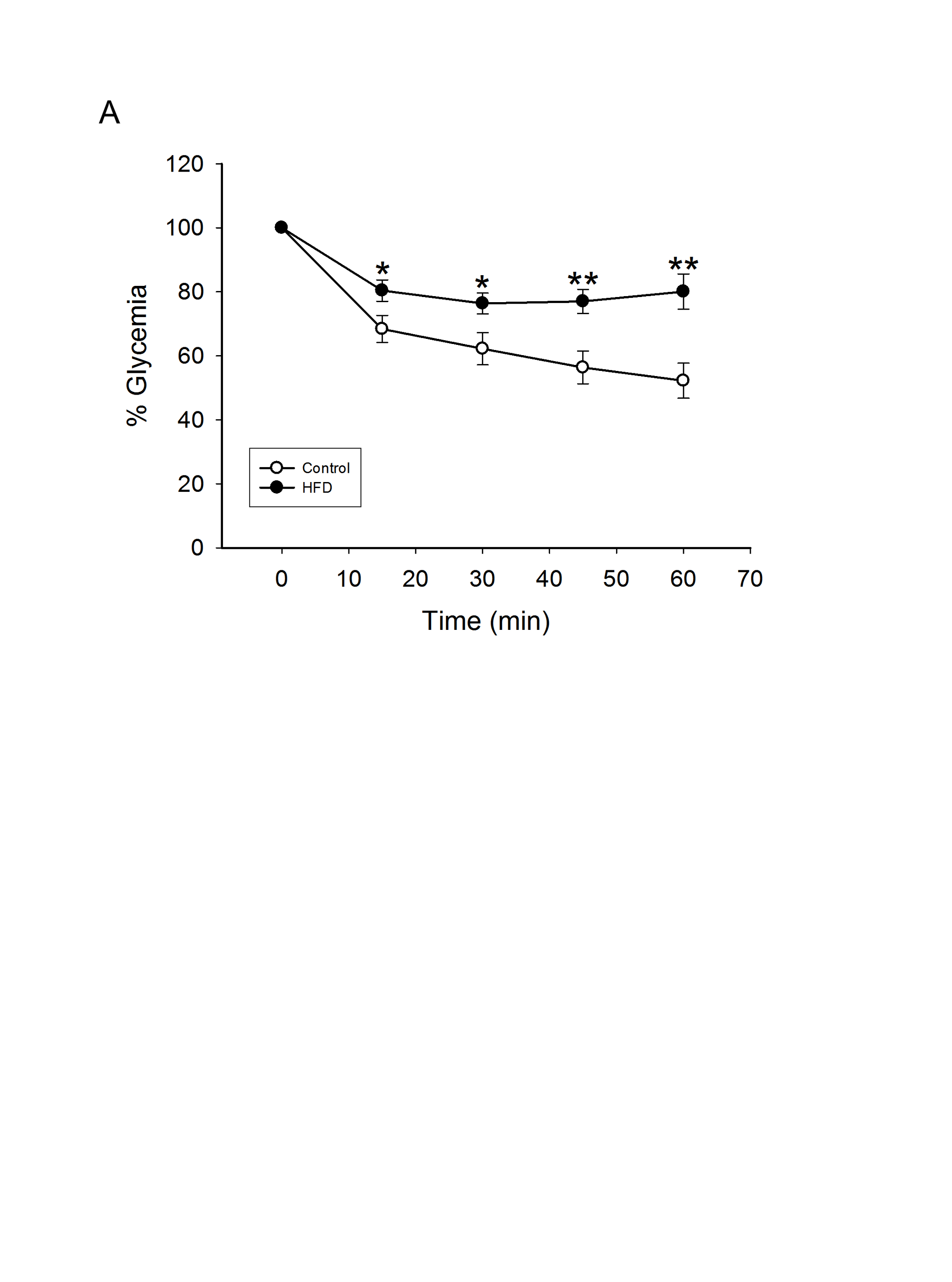

Supplement: Figure S3 — Insulin sensitivity is decreased in HFD animals compared to control at 28 weeks of age. ipITT was performed in animals fed with HFD or chow diet during 24 weeks (n≥11 animals from ≥6 litters). Data are expressed in mean±SEM. Significance P<0.05 by t-test. (TIF) [file pone.0100214.s003.tif]

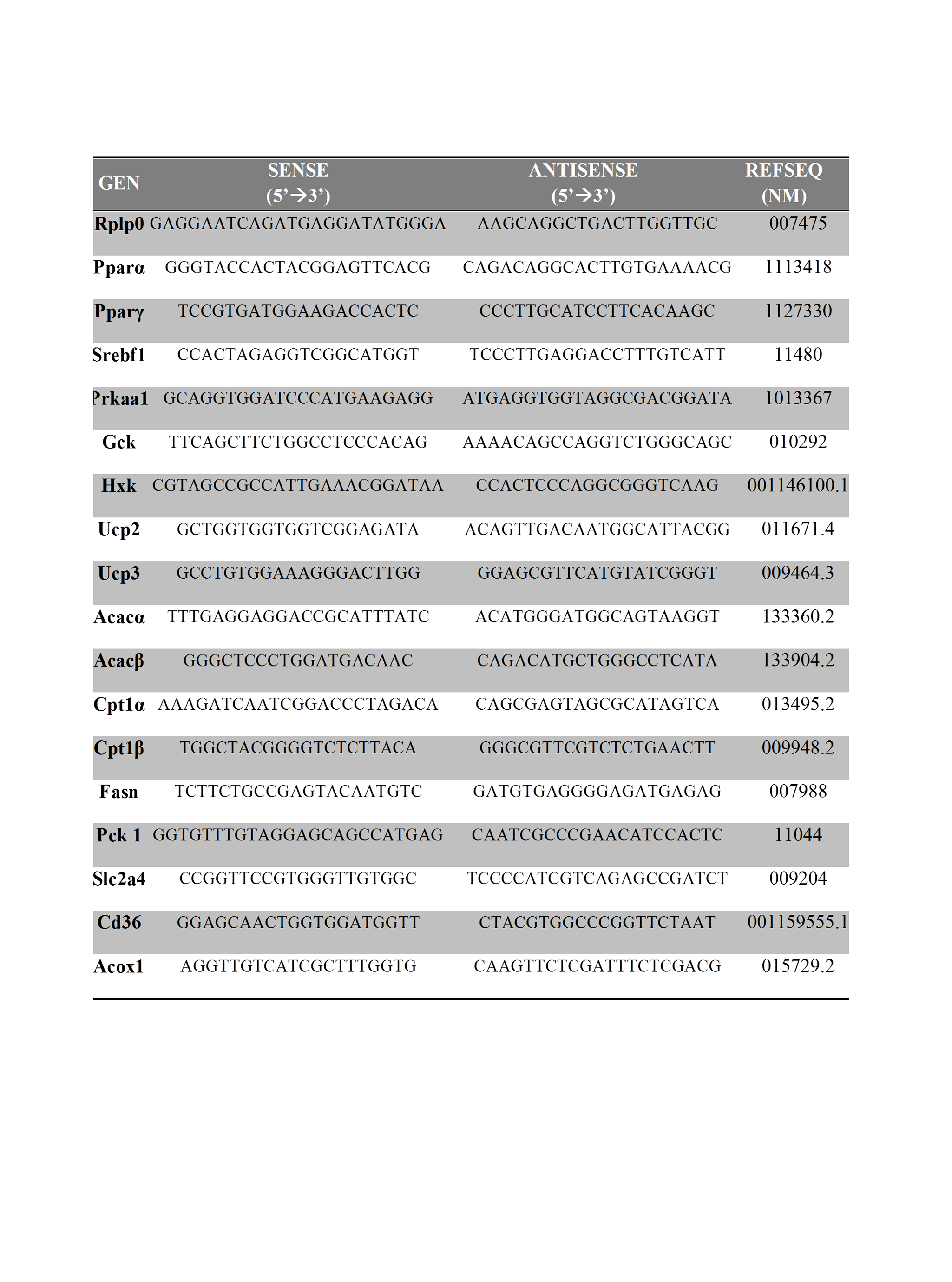

Supplement: Table S1 — Real time PCR primers. (TIF) [file pone.0100214.s004.tif]
